# Volumetric Magnetic Resonance Imaging Study of Brain and Cerebellum in Children with Cerebral Palsy

**DOI:** 10.1155/2016/5961928

**Published:** 2016-08-04

**Authors:** Piotr Kułak, Elżbieta Maciorkowska, Elżbieta Gościk

**Affiliations:** ^1^Department of Pediatric Radiology, Medical University of Białystok, 15-274 Białystok, Poland; ^2^Department of Developmental Age Medicine and Pediatric Nursing, Medical University of Białystok, 15-296 Białystok, Poland

## Abstract

*Introduction*. Quantitative magnetic resonance imaging (MRI) studies are rarely used in the diagnosis of patients with cerebral palsy. The aim of present study was to assess the relationships between the volumetric MRI and clinical findings in children with cerebral palsy compared to control subjects.* Materials and Methods*. Eighty-two children with cerebral palsy and 90 age- and sex-matched healthy controls were collected.* Results*. The dominant changes identified on MRI scans in children with cerebral palsy were periventricular leukomalacia (42%) and posthemorrhagic hydrocephalus (21%). The total brain and cerebellum volumes in children with cerebral palsy were significantly reduced in comparison to controls. Significant grey matter volume reduction was found in the total brain in children with cerebral palsy compared with the control subjects. Positive correlations between the age of the children of both groups and the grey matter volumes in the total brain were found. Negative relationship between width of third ventricle and speech development was found in the patients. Positive correlations were noted between the ventricles enlargement and motor dysfunction and mental retardation in children with cerebral palsy.* Conclusions*. By using the voxel-based morphometry, the total brain, cerebellum, and grey matter volumes were significantly reduced in children with cerebral palsy.

## 1. Introduction

Advances in neuroimaging provide unique opportunities to evaluate brain structure, biochemistry, and function [1].

Parents and clinicians concerned about high-risk infants and children with motor delay or cerebral palsy seek information on cause, treatment, and prognosis. Used in combination with history and examination, neuroimaging studies can improve diagnosis and management. In children with cerebral palsy syndromes including spastic diplegia, quadriplegia, hemiplegia, and extrapyramidal movement disorders, conventional magnetic resonance imaging (MRI) has become an important determinant of diagnosis, management, and prognosis [2]. Conventional MRI reveals anatomical features of the brain and is typically used to quantify brain tissue volume and shape.

Currently, it is now widely accepted that most cerebral palsy is not the result of intrapartum hypoxia [3, 4]. Lesions responsible for periventricular leukomalacia and posthemorrhagic porencephaly are considered to occur early in the third trimester. Both vascular and intrinsic metabolic factors are thought to be responsible for the localization of periventricular leukomalacia. In patients with cerebral palsy, congenital abnormalities are often found in MRI [5, 6].

Ventricular enlargement is common in preterm and low-birth-weight infants. Furthermore, ventricular enlargement on cranial ultrasound in children was found to be significant risk factor for cerebral palsy [7]. Enlargement of the ventricles may occur for a number of reasons, such as loss of brain volume (infection, hypoxia, and infarction) or impaired outflow or absorption of cerebrospinal fluid from the ventricles.

It was also demonstrated that ventricular dilatation increases the risk of poor development of children born at term. And Evans' index (ventricular-brain ratio) above 0.35 is a sensitive measure of developmental impairment [8].

Conventional MRI is currently recommended as a standard evaluation in children with cerebral palsy where the aetiology has not been established [9, 10]. However, quantitative MRI studies are rarely used in the diagnosis of patients with cerebral palsy [11, 12]. Voxel-based morphometry (VBM) as an automated technique is more sensitive than conventional MRI in investigating the structural changes of the whole brain [11]. The recent MRI studies have employed VBM to detect regional grey matter and white matter volume abnormalities in children with cerebral palsy [13, 14].

However, previous studies mainly focused on patients with cerebral palsy who exhibited abnormal findings in conventional MR particularly in white matter changes [15–17]. Until now, only few studies have investigated grey matter changes in the brain in children with cerebral palsy [18].

The first objective was to detect grey matter changes in the brain in children with cerebral palsy using the VBM technique. The second one was to investigate the relationships between the volumetric MRI and clinical findings in children with cerebral palsy.

## 2. Materials and Methods

### 2.1. Study Design

We retrospectively evaluated MRI findings and medical data of children with cerebral palsy referred to Department of Pediatric Radiology in Białystok, Poland, from imaging studies from January 2006 to December 2012. All children with cerebral palsy and control children were born to mothers living in the Podlaskie region.

### 2.2. Subjects

The present study included 82 children with cerebral palsy. Of these children, 30 had spastic diplegia, 27 had spastic tetraplegia, and 25 had spastic hemiplegia. A group of 96 healthy right-handed children matched for age and gender were recruited as a comparison group. All subjects were free from neurological or psychiatric diseases, they had normal intellectual development, and their brain MRI scans were normal. Children with postnatal meningitis, encephalitis, trauma, intra-axial or extra-axial tumors other than small prechiasmatic and chiasmatic optic nerve gliomas, and metabolic or degenerative disorders were excluded from the study.

### 2.3. Motor Function

Each child was classified according to the Gross Motor Function Classification System (GMFCS) [19].

### 2.4. Mental Development

All the children in this group had 1 or more formal psychological assessments (the typical Wechsler Intelligence Scale for Children, Polish version). Mental development was divided into small delay, 70 to 84 IQ; moderate delay, 50 to 69 IQ; and severe delay, <50 IQ. Normal children have an IQ > 90. All 96 healthy subjects had normal intelligence.

### 2.5. MRI

All MRI scans were obtained using a 0.35-T MR scanner (Siemens, Germany) with the use of a standard circular polarized head coil. The images were assessed by a neuroradiologist (Elżbieta Gościk). A set of sagittal T1-weighted images were acquired with the following scan parameters: a time repetition (TR) of 20 ms, a time echo (TE) of 8.9 ms, and a flip angle of 45°. Each volume consisted of between 124 and 128 sagittal slices, and each slice had dimensions of 256 × 256 pixels. Voxel's dimensions were 0.9 × 0.9 × 5 mm. Routinely, brain data sets were saved and stored in a standard DICOM format controlled by a Kodak Direct View OSM (Eastman Kodak Company, USA) storing system. For postacquisition processing of MR images, the DICOM files were transferred into a Windows controlled desktop PC, where they were processed using specialized image data processing software.

### 2.6. Image Analysis

**Figure 1:**
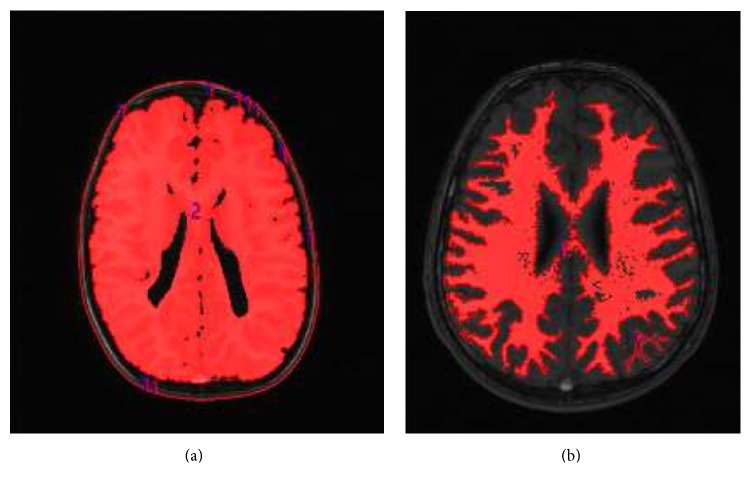
(a) An image of whole brain by the program Analyze 11 (own source). (b) An image of the white matter of brain by the program Analyze 11 (own source).

Analyze 11 Biomedical Imaging Software (AnalyzeDirect, Overland Park, KS, USA) was used to estimate the total grey matter of the brain and the brain and cerebellum volumes of each subject. The collected original MRI DICOM volumetric data were then converted to internal volumetric AVW format. When analyzing the images, Analyze 11 uses a variety of volume rendering algorithms, including volumetric compositing, depth shading, shading colors, maximal intensity shade of grey, the entire volume, and particular volume voxels per pixel in the image DICOM. Voxel (volumetric element) is three-dimensional graphics smallest element of space, in a sense equivalent to two-dimensional pixel graphics. Using a voxel image shows a three-dimensional array as, for example, 512 × 512 × 512 voxels. This software also allows image display at three levels, that is, transverse, frontal, and sagittal view of the volume (Figure 1).

Prior to voxel-based morphometry preprocessing, nonbrain tissues surrounding the entire brain in each image were manually removed using the Analyze 11 software to maximize sensitivity in data analysis [20]. The global volumes of grey matter, white matter, and cerebrospinal fluid for each scan were calculated from the maps of the raw (nonnormalized) MRI. The volume of each tissue class was estimated as the total number of voxels multiplied by the voxel size. Whole-brain tissue volume was calculated by summing the grey matter, white matter, and cerebrospinal fluid volumes for each subject. However, in this study, we presented only data of the total brain, cerebellum, and the grey matter volumes. We did not analyze the white matter volumes.

### 2.7. Definitions

Cerebral palsy was defined as a group of disorders of the development of movement and posture, causing activity limitation, that are attributed to nonprogressive disturbances that occurred in the developing fetal or infant brain [21]. Cerebral palsy was classified as spastic tetraplegia (spasticity of all four limbs and of about equal involvement) and spastic diplegia (spasticity of lower limbs more pronounced than upper limbs). Hemiplegic cerebral palsy refers to one arm and one leg affected on either the right or left side of the body. Epilepsy was defined as a separate occurrence of two or more apparently unprovoked seizures. Prematurity was defined by the World Health Organization as an infant with a gestation of less than 37 weeks from the first day of the last menstrual period. Asphyxia is defined as an Apgar score ≤4.

### 2.8. Statistical Analysis

Descriptive statistics were used to examine each study variable individually. The differences between the groups were determined by the parametric* t-*test and nonparametric statistical tests: Wilcoxon signed-rank test and Chi-square test where appropriate. Linear regression was used to measure the dependence regarding age, gender, Apgar score, mental retardation, and Gross Motor Function Classification System of cerebral palsy and grey matter volume. All *P* values were two-tailed. Statistical significance was defined as *P* < 0.05. Statistics were calculated using Statistica 10.0.

### 2.9. Ethics

The study was approved by the ethics committee of the Medical University of Białystok, Poland (number R-I-002/244/2009). Consent was not obtained from parents of children. Patient records were anonymized and deidentified prior to analysis.

## 3. Results

In total, 52 boys and 30 girls, with age range of 3–17 years, mean age of 10.9 ± 6.9 years, and cerebral palsy on MRI, were included. More than half of the children (48) were born at term and 34 were born preterm. Thirty patients experienced birth asphyxia (≤4 Apgar score). In the control group, 47 girls and 43 boys, with age range of 1–18 years and mean age of 12.4 ± 4.3 years, on MRI were included. The proportions of males and females between the groups did not differ significantly. Details are shown in Table 1.

### 3.1. MRI Findings

This study's sample of 82 patients with cerebral palsy showed a high rate (80%) of positive findings. In our study, 19 patients (20%) had normal MRIs. The dominant changes identified on MRI scans in children with cerebral palsy were periventricular leukomalacia (PVL) (42.4%), posthemorrhagic hydrocephalus (20.7%), and ventriculomegaly of lateral ventricles (9.7%). These changes were found in 60 patients with cerebral palsy. In other cases, cortical-subcortical atrophy (9.7%), porencephaly (6.5%), Dandy-Walker syndrome (2.2%), and corpus callosum hypoplasia (2.2%) were found. In single cases, corpus callosum agenesis, schizencephaly, and pachygyria were noted. All porencephaly cases were observed in patients with spastic hemiplegia.

All cortical-subcortical atrophy and congenital brain malformations were noted in patients with spastic tetraplegia. Sixteen patients with cerebral palsy had normal MRI scan results. Normal MRI results were found in patients with spastic diplegia.

### 3.2. Volumetric MRI Findings

The total grey matter volume of the brain was significantly (*P* < 0.028) reduced in the patient group compared with controls. Details are shown in Table 2.

The total brain and cerebellum volumes were significantly (*P* < 0.001) reduced in children with cerebral palsy compared to the control subjects. The lateral ventricles and third-ventricle widths were significantly (*P* < 0.001) larger in children with cerebral palsy compared to controls (Table 2).

Significant difference (*P* < 0.001) was found in Evans' index between children with cerebral palsy (0.34 ± 0.23) and the control group (0.27 ± 0.21).

### 3.3. MRI Correlations

Linear regression demonstrated a positive relationship (*r* = 0.265; *P* = 0.011) between the total brain volume and the age in children with cerebral palsy (Table 3). Similar correlation (*r* = 0.289; *P* = 0.004) was also noted in the control group.

Age of children with cerebral palsy was positively correlated with the total grey matter volume of brain (*r* = 0.248; *P* = 0.016). Similar correlation (*r* = 0.323; *P* = 0.001) was also found in the control subjects (Table 4).

No correlations between the total grey matter volume of brains and gender, Apgar score, birth weight, motor development, mental development, and epilepsy in both groups were noted.

No significant correlations between the total cerebellum volume and the tested variables in both groups were found. Details are not shown.

Significant correlation (*r* = 0.205; *P* = 0.049) between the width of lateral ventricles and GMFCS and mental retardation in children with cerebral palsy was found (Table 5).

No significant correlations between the width of lateral ventricles and the tested variables in the control group were found.

Negative correlation (*r* = −0.780; *P* = 0.0002) between third-ventricle width and speech development in children with cerebral palsy was found. A positive relationship (r = 0.221; *P* = 0.033) between third-ventricle width and mental retardation was found. Data are shown in Table 6.

## 4. Discussion

We demonstrated significant abnormalities with MRI in 80% of children with cerebral palsy. The dominant changes identified on MRI scans of patients were PVL. Our findings are comparable with the results of previous reports [4, 6, 22–24].

In the current study, we found significant grey matter volume reduction (the total grey matter volume of the brain and cerebellum) in children with cerebral palsy compared to controls. The grey matter volume reductions in the cerebral palsy patients suggest neuronal degeneration and damage. Our findings are in agreement with those of previous studies [18, 22].

The pathogenesis of grey matter lesions in PVL is likely due to the same phenomena implicated in white matter lesions [22]. The pathogenesis of PVL likely involves cerebral ischemia-reperfusion. Moreover, we found a significant correlation between the width of the lateral ventricles and motor function in the GMFCS scale and mental retardation in children with cerebral palsy.

Typical preterm brain injuries include PVL and posthemorrhagic porencephaly [4, 6, 22, 23]. PVL usually occurs between gestational weeks 28 and 34 and is caused by an ischemic process in the watershed zone that exists in the periventricular white matter of the immature brain. The MRI features of PVL include a reduced quantity of periventricular white matter, periventricular gliosis, and ventriculomegaly with an irregular outline of the lateral ventricles [4, 16, 17, 24]. In the present study, nearly 41% of patients with cerebral palsy were born prematurely and had typical PVL, and PVL (42%) was the dominant lesion in MRI findings. Furthermore, in the conventional MRI, cortical-subcortical lesions were seen in 9.7% of patients. Meanwhile, in the volumetric MRI, significantly reduced grey matter volume was found in children with cerebral palsy compared with controls. Such results suggest that subtle structural injuries, supposedly related to motor dysfunction, may exist in these children [14]. In the present study, normal MRI findings were found only in patients with spastic diplegia.

The conventional understanding has maintained that cerebral white matter in the premature brain is especially vulnerable to hypoxia-ischemia with relative sparing of the grey matter and that grey matter injuries dominate overwhelmingly in older infants, children, and adults [25]. However, quantitative volumetric MRI studies of premature infants have demonstrated reduced volumes of the cerebral cortex [26], thalamus, basal ganglia [27], and hippocampus [28].

In a neuropathological study of premature infants, Pierson et al. [22] found that grey matter lesions occurred in at least a third of PVL cases, suggesting that white matter injuries generally do not occur in isolation and that the term* perinatal panencephalopathy *may better describe the scope of such neuropathology. Yin et al. [29] have reported subcortical lesions and cortical atrophy in infants born at or after 35-36 weeks of gestation. In the current study, such changes occurred in 9.7% of patients, all of whom were born at term.

Grey matter injuries such as neuronal degeneration, gliosis, and subplate neuronal damage are known to be commonly associated with PVL following prenatal hypoxic-ischemic insult [21, 30]. These neuronal injuries reduce regional volume, and neuronal damage occurs close to the predilection sites of white matter injury, involving predominantly the posterior brain region [30]. Our study found that the volumes of the total brain and cerebellum, as well as the widths of the lateral and third ventricles, differed significantly between children with cerebral palsy and controls. Furthermore, 41% of these children were born prematurely. The enlarged lateral and third ventricles are thought to be due to subcortical tissue loss associated with prematurity [28].

Cerebral ventricular enlargement occurs in numerous conditions, including normal aging, traumatic brain injury, and hypoxia, and has been suggested to predict neuronal loss [31]. In our study, ventricular enlargement appeared in patients with cerebral palsy and was correlated with mental retardation and delayed speech development. Our results are in agreement with those of Lowe et al. [32], in which negative correlations between the lateral ventricles and frontal and hippocampal volumes were reported in preterm infants.

A ventricular-brain ratio exceeding 0.35 is a sensitive measure of developmental impairment (8.34). In the present study, a significant difference emerged in the Evans index between children with cerebral palsy (0.34 ± 0.23) and the controls (0.27 ± 0.21).

Numata et al. [31] investigated the association between MRI patterns and motor function, epileptic episodes, and mental development in patients born at term with spastic diplegia. MRI included normal findings (41.9%), PVL, hypomyelination, and porencephaly or periventricular venous infarction. Moreover, the frequency of patients at GMFCS levels III–V and with intellectual disability did not differ between patients with normal and abnormal MRI findings. Patients with normal MRI findings had significantly fewer epileptic episodes than those with abnormal ones. In a systematic review, Arnfield et al. [33] reported a relationship between the type of brain lesion on MRI and gross motor function in children with cerebral palsy. These results are consistent with the findings of the present study.

An association with preterm birth or low birth weight has been found in several specific types of defects of the central nervous system, including anencephaly, hydrocephaly, microcephaly, encephalocele, spina bifida, microphthalmia, and cleft lip or palate [34].

Brain malformations originating from intrauterine maldevelopment may underlie neurologic impairment seen in children with cerebral palsy, who have a greater incidence of congenital brain malformations [9, 35, 36]. It is estimated that roughly 10% of children with cerebral palsy have different brain malformations. In the present study, we found congenital brain malformations (e.g., corpus callosum agenesis, schizencephaly, lissencephaly, and pachygyria) in 7.6% of the sample.

The MRI findings may help us to understand not only the type of lesion but also the timing of insult. The early detection of brain abnormalities in children with cerebral palsy may help in their prognosis and in the introduction of appropriate therapies (physical therapy or language therapy).

*Limitations*. Our population was heterogeneous in regard to the type of cerebral palsy. Second, this is a retrospective study. Third, we analyzed MRI scans with a low resolution. Replication studies within specific age groups and/or larger sample sizes and better MRI resolution will be important for establishing the generalizability of the present findings.

## 5. Conclusion

By using the voxel-based morphometry, the total brain, cerebellum, and grey matter volumes were significantly reduced in children with cerebral palsy. The grey matter volume reductions in the cerebral palsy patients suggest neuronal degeneration and damage. And ventricular enlargement was found to be significantly correlated with motor dysfunction and both speech and mental retardation in these patients. Our findings offer a deeper insight into the pathophysiological mechanisms of cerebral palsy. The voxel-based morphometry of brain in children with cerebral palsy may help in prognosis and in the introduction of rehabilitation of motor impairment or speech and language therapy.

## Figures and Tables

**Table 1:** Clinical data of children cerebral palsy and controls.

Data	Cerebral palsy (*n* = 82)	Controls (*n* = 90)	*P* value
Age, y (range, 7–15)	10.9 ± 6.9	12.4 ± 4.3	NS
Gender			
Male	52	43	NS
Female	30	47	NS
Vaginal birth	61	68	NS
Cesarean birth	21	24	NS
Gestation, wk	35.9 ± 4.2	39.2 ± 1.2	<0.001
Delivery			
Term	48	85	NS
Preterm <37 wk	34	5	<0.001
Apgar score (1–10)	5.9 ± 3.3	9.1 ± 1.5	<0.001
Asphyxia	30	1	<0.001
Birth weight, g	2615.8 ± 935.1	3343.2 ± 497.6	<0.001
Low birth weight (<2500 g)	22	5	0.0022
Normal birth weight (>2500 g)	60	85	NS
GMFCS^∧^			
Level 1	0	90	
Level 2	20	0	
Level 3	25	0	
Level 4	24	0	
Level 5	13	0	
Mental development			
Normal	43	90	0.0098
Small delay	17	0	<0.001
Mild	12	0	<0.001
Severe	10	0	0.0032
Epilepsy	24	0	<0.001

Chi-square test; NS: not significant; ∧: not compared.

**Table 2:** Mean values of the total grey matter volume of brain, brain volume, cerebellum volume, and the widths of the lateral ventricles and third ventricle in children with cerebral palsy (*n* = 82) and the control group (*n* = 90).

Variable	Mean	Min.	Max.	SD	*P* value
Total grey matter volume of brain (mm^3^)					
Cerebral palsy	8,986.31	320.18	1,9458.62	5,283.03	0.028
Control	10,803.78	593.10	1,8954.58	5,492.06	
Total brain volume (mm^3^)					
Cerebral palsy	1,1943.38	1,911.83	2,1612.53	5,649.39	<0.001
Control	14,800.77	3,314.16	2,3411.76	5,611.59	
Total cerebellum volume (mm^3^)					
Cerebral palsy	919.65	554.37	1,721.19	214.10	<0.001
Control	1,426.54	748.51	2,506.12	349.84	
The lateral ventricles width (mm)					
Cerebral palsy	37.58	25.93	81,31	9.29	<0.001
Control	33.68	28.29	40,51	2.41	
Third-ventricle width (mm)					
Cerebral palsy	5.26	1.78	17.46	3.25	<0.001
Control	2.59	1.52	5.97	0.93	

Wilcoxon signed-rank test.

**Table 3:** Correlations between total brain volume and age, gender, gestational age, Apgar score, birth weight, GMFCS, motor and speech development, mental retardation, and epilepsy in children with cerebral palsy (*n* = 82) and controls (*n* = 90).

Variable	*r* value	*P* value
Total brain volume versus age		
Cerebral palsy	0.265	0.011
Control	0.289	0.004
Total brain volume versus male sex		
Cerebral palsy	−0.085	NS
Control	−0.091	
Total brain volume versus female sex		
Cerebral palsy	−0.037	NS
Control	−0.081	
Total brain volume versus gestational age		
Cerebral palsy	0.100	NS
Control	0.085	
Total brain volume versus Apgar score		
Cerebral palsy	0.1106	NS
Control	0.0486	
Total brain volume versus birth weight		
Cerebral palsy	0.125	NS
Control	−0.011	
Total brain volume versus GMFCS		
Cerebral palsy	0.0576	NS
Control	∧	
Total brain volume versus siting		NS
Cerebral palsy	0.1477	
Control	−0.123	
Total brain volume versus standing		NS
Cerebral palsy	0.115326	
Control	−0.04719	
Total brain volume versus walking		NS
Cerebral palsy	0.127084	
Control	−0.10585	
Total brain volume versus speech		NS
Cerebral palsy	0.150530	
Control	0.020021	
Total brain volume versus mental retardation		NS
Cerebral palsy	−0.14620	
Control	0.288966	
Total brain volume versus epilepsy		NS
Cerebral palsy	0.027474	
Control	0.017008	

*r*: Spearman rank sum correlation coefficient; NS: not significant. ∧: not tested for controls.

**Table 4:** Correlations between the total grey matter volume of brain and age, gender, gestational age, Apgar score, birth weight, GMFCS, motor and speech development, mental retardation, and epilepsy in children with cerebral palsy (*n* = 82) and controls (*n* = 90).

Variable	*r* value	*P* value
Grey matter volume versus age		
Cerebral palsy	0.248	0.017
Control	0.323	0.001
Grey matter volume versus male sex		NS
Cerebral palsy	0.013	
Control	0.057	
Grey matter volume versus female sex		NS
Cerebral palsy	−0.024	
Control	0.048	
Grey matter volume versus gestational age		NS
Cerebral palsy	−0.023	
Control	0.028	
Grey matter volume versus Apgar score		NS
Cerebral palsy	0.044	
Control	0.038	
Grey matter volume versus birth weight		NS
Cerebral palsy	−0.005	
Control	−0.032	
Grey matter volume versus GMFCS		
Cerebral palsy	0.036	NS
Control	∧	
Grey matter volume versus siting		NS
Cerebral palsy	0.197	
Control	−0.011	
Grey matter volume versus standing		NS
Cerebral palsy	0.098	
Control	0.068	
Total brain volume versus walking		NS
Cerebral palsy	0.085	
Control	−0.024	
Grey matter volume versus speech development		
Cerebral palsy	0.090	
Control	0.117	NS
Grey matter volume versus mental retardation		NS
Cerebral palsy	−0.045	
Control	0.323	
Grey matter volume versus epilepsy		NS
Cerebral palsy	0.022	
Control	−0.047	

*r*: Spearman rank sum correlation coefficient; NS: not significant; ∧: not tested for controls.

**Table 5:** Correlations between the lateral ventricles widths of brain and age, gender, gestational age, Apgar score, birth weight, GMFCS, motor and speech development, mental retardation, and epilepsy in children with cerebral palsy (*n* = 82) and controls (*n* = 90).

Variable	*r* value	*P* value
The lateral ventricles width versus age		
Cerebral palsy	0.078	NS
Control	0.579	
The lateral ventricles width versus male sex		
Cerebral palsy	0.048	NS
Control	0.078	
The lateral ventricles width versus female sex		
Cerebral palsy	0.060	NS
Control	−2.639	
The lateral ventricles width versus gestational age		
Cerebral palsy	0.067	NS
Control	0.134	
The lateral ventricles width versus Apgar score		
Cerebral palsy	−0.040	NS
Control	−0.081	
The lateral ventricles width versus birth weight		
Cerebral palsy	−0.098	NS
Control	−2.270	
The lateral ventricles width versus GMFCS		
Cerebral palsy	0.314	0.002
Control	∧	
The lateral ventricles width versus siting		
Cerebral palsy	0.136	NS
Control	−0.488	
The lateral ventricles width versus standing		
Cerebral palsy	−0.092	NS
Control	−0.061	
The lateral ventricles width versus walking		
Cerebral palsy	−0.068	NS
Control	−0.174	
The lateral ventricles width versus speech development		
Cerebral palsy	−0.154	NS
Control	−0.044	
The lateral ventricles width versus mental retardation		
Cerebral palsy	0.205	0.049
Control	−1.048	NS
The lateral ventricles width versus epilepsy		
Cerebral palsy	0.088	NS
Control	0.579	

*r*: Spearman rank sum correlation coefficient; NS: not significant; ∧: not tested for controls.

**Table 6:** Correlations between the third-ventricle widths of brain and age, gender, gestational age, Apgar score, birth weight, GMFCS, motor and speech development, mental retardation, and epilepsy in children with cerebral palsy (*n* = 82) and controls (*n* = 90).

Variable	*r* value	*P* value
Third-ventricle width versus age		
Cerebral palsy	−0.106	NS
Control	0.177	
Third-ventricle width versus male sex		
Cerebral palsy	0.049	
Control	−0.003	NS
Third-ventricle width versus female sex		
Cerebral palsy	−0.004	
Control	0.003	NS
Third-ventricle width versus gestational age		
Cerebral palsy	−0.046	
Control	−0.055	NS
Third-ventricle width versus Apgar score		
Cerebral palsy	−0.021	
Control	0.126	NS
Third-ventricle width versus birth weight		
Cerebral palsy	−0.077	
Control	−0.090	NS
Third-ventricle width versus GMFCS		
Cerebral palsy	0.178	0.088
Control	∧	
Third-ventricle width versus sitting		
Cerebral palsy	−0.066	
Control	−0.136	NS
Third-ventricle width versus standing		
Cerebral palsy	−0.191	
Control	−0.088	NS
Third-ventricle width versus walking		
Cerebral palsy	−0.198	
Control	−0.148	NS
Third-ventricle width versus speech development		
Cerebral palsy	−0.378	0.0002
Control	−0.094	
Third-ventricle width versus mental retardation		
Cerebral palsy	0.221	0.0334
Control	0.177	
Third-ventricle width versus epilepsy		
Cerebral palsy	0.199	NS
Control	−0.003	

*r*: Spearman rank sum correlation coefficient; NS: not significant; ∧: not tested for controls.
